# The Development of a Text Messaging Platform to Enhance a Youth Diabetes Prevention Program: Observational Process Study

**DOI:** 10.2196/45561

**Published:** 2024-05-29

**Authors:** Manali Sapre, Cordelia R Elaiho, Rena Brar Prayaga, Ram Prayaga, Jeremy Constable, Nita Vangeepuram

**Affiliations:** 1 New York University Langone New York, NY United States; 2 Medical College of Wisconsin Milwaukee, WI United States; 3 Department of General Pediatrics Icahn School of Medicine at Mount Sinai New York, NY United States; 4 mPulse Mobile Los Angeles, CA United States; 5 Community Action Board Icahn School of Medicine at Mount Sinai New York, NY United States; 6 Department of Population Health Science and Policy Icahn School of Medicine at Mount Sinai New York, NY United States; 7 Institute for Health Equity Research Icahn School of Medicine at Mount Sinai New York, NY United States

**Keywords:** community-based participatory research, youth, diabetes prevention, peer education, mobile health technology, SMS text messaging, mobile phone, artificial intelligence, AI

## Abstract

**Background:**

Approximately 1 in 5 adolescents in the United States has prediabetes, and racially and ethnically minoritized youths are disproportionately impacted. Unfortunately, there are few effective youth diabetes prevention programs, and in-person interventions are challenging because of barriers to access and engagement.

**Objective:**

We aimed to develop and assess the preliminary feasibility and acceptability of a youth-informed SMS text messaging platform to provide additional support and motivation to adolescents with prediabetes participating in a diabetes prevention workshop in East Harlem, New York City, New York, United States. We collaborated with our youth action board and a technology partner (mPulse Mobile) to develop and pilot-test the novel interactive platform.

**Methods:**

The technology subcommittee of our community action board (comprising youths and young adults) used the results from focus groups that we had previously conducted with youths from our community to develop 5 message types focused on healthy eating and active living: goal setting, behavior tracking, individually tailored guidance, motivational messages, and photo diary. We used an iterative process to develop and pilot the program with our internal study team, including youths from our community action board and mPulse Mobile developers. We then conducted a pilot of the 12-week SMS text messaging program with 13 youths with prediabetes.

**Results:**

Participants (aged 15-21 years; 10/13, 77% female; 3/10, 23% Black and 10/13, 77% Hispanic or Latinx) received an average of 2 automated messages per day. The system correctly sent 84% (2231/2656) of the messages at the time intended; the remaining 16% (425/2656) of the messages were either sent at the incorrect time, or the system did not recognize a participant response to provide the appropriate reply. The level of engagement with the program ranged from 1 (little to no response) to 5 (highly responsive) based on how frequently participants responded to the interactive (2-way) messages. Highly responsive participants (6/13, 46%) responded >75% (1154/1538) of the time to interactive messages sent over 12 weeks, and 69% (9/13) of the participants were still engaged with the program at week 12. During a focus group conducted after program completion, the participants remarked that the message frequency was appropriate, and those who had participated in our in-person workshops reflected that the messages were reminiscent of the workshop content. Participants rated goal setting, behavior tracking, and tailored messages most highly and informed planned adaptations to the platform. Participants described the program as: “interactive, informative, enjoyable, very convenient, reliable, motivational, productive, and reflective.”

**Conclusions:**

We partnered with youths in the initial content development and pilot testing of a novel SMS text messaging platform to support diabetes prevention. This study is unique in the triple partnership we formed among researchers, technology experts, and diverse youths to develop a mobile health platform to address diabetes-related disparities.

## Introduction

### Background

The rise in the prevalence of type 2 diabetes mellitus (T2DM) and prediabetes (a condition in which blood glucose levels are higher than normal but not high enough to diagnose diabetes) among youths has been widely reported as a growing epidemic. In the United States, approximately 1 in 5 adolescents has prediabetes [1]. A 2016 consensus report by the American Diabetes Association highlighted racial and ethnic disparities in youth-onset T2DM, with a prevalence 4 times higher among Hispanic or Latinx youths and 5 times higher among Black youths than among their non-Hispanic White peers [2]. There is evidence that youth-onset T2DM progresses more quickly and results in earlier complications than adult-onset T2DM [2]. However, it is promising that lifestyle interventions have proven effective in preventing or reversing the progression of prediabetes to T2DM. A large randomized controlled trial in adults showed that a lifestyle intervention was twice as effective as blood glucose–lowering medications in preventing diabetes among adults considered to be at high risk [3]. Another study found that adolescents with obesity who maintained their weight and BMI were more likely to revert from impaired to normal blood glucose tolerance than those who gained weight [4].

Unfortunately, there are few effective prevention programs for racially and ethnically minoritized youths who are disproportionately affected by this disease [5,6]. The population in our community (Harlem in New York City, New York, United States) is largely Hispanic or Latinx and Black. Almost half of all school-age children and a third of high school students living in this community are overweight or obese [7,8]. Given the strong association between obesity and prediabetes among adolescents [1], there is a pressing need for effective diabetes screening and prevention programs in communities such as ours. To address this gap, we developed Teen Help Educate to Eliminate Diabetes (HEED), a 12-week peer-led diabetes prevention intervention aimed at improving lifestyle and metabolic risk factors among youths with prediabetes. We developed this intervention using community-based participatory research (CBPR), a collaborative approach that incorporates the values of both community and academic stakeholders in the research process [9]. Our preliminary results showed that most of the adolescents who completed >50% of the program no longer had prediabetes at the 3-month follow-up and that workshop participants had an improvement in physical activity self-efficacy and health behaviors such as portion control [10].

Despite these promising results, it remains a challenge to impact youths through in-person interventions because of barriers to access and engagement [11]. One potential solution is to deliver interventions via virtual platforms to reach more youths and address logistical barriers such as scheduling, transportation, and competing priorities [11]. The Pew Research Center reported that smartphone access among teens in the United States increased dramatically from 73% in 2014-2015 to 95% in 2018 [12,13]. Smartphone ownership is almost ubiquitous among teens, regardless of gender, race, ethnicity, or socioeconomic background [12]. In fact, teens from lower-income households are more likely to use their phone to access social networks and health information than those from higher-income households [12]. Thus, mobile health (mHealth) interventions are even more promising among adolescents who are most at risk and who also face socioeconomic barriers affecting access to prevention programs.

### Objectives

In this study, our aim was to develop an mHealth SMS text messaging intervention as a potential avenue to broaden the impact of Teen HEED. To do this, we partnered with mPulse Mobile, an mHealth technology company, as well as our youth action board to develop a novel SMS text messaging tool to support diabetes prevention efforts targeting youths with prediabetes in East Harlem. In this paper, we present the participatory process we used in collaboration with youths and our technology partners to iteratively develop and refine the texting program and examine feasibility and acceptability data from early pilot and usability testing.

## Methods

**Figure 1:**
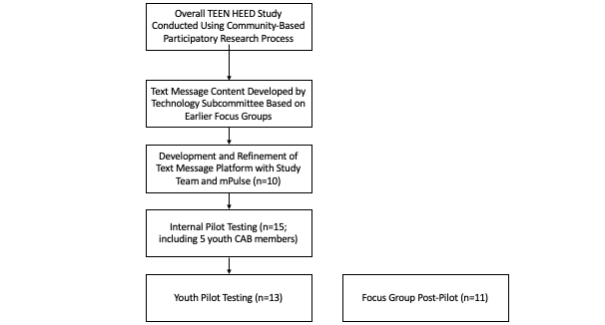
CONSORT (Consolidated Standards of Reporting Trials) flow diagram. CAB: community action board; HEED: Help Educate to Eliminate Diabetes.

The CONSORT (Consolidated Standards of Reporting Trials) flow diagram, presented in Figure 1, summarizes the various phases of the study, which are described in more detail in the following subsections.

### Preliminary Studies

As a first step to better understand the feasibility of an mHealth lifestyle intervention for youths in the East Harlem community, we conducted 6 focus groups with >50 young people recruited through our community partners [10]. We examined social and environmental factors that may impact lifestyle, barriers to healthy behaviors, and the ways in which mHealth may be used as part of diabetes prevention efforts. Most of the teens in our focus groups preferred SMS text messaging over social media and mHealth apps to deliver healthy lifestyle content [10]. The teens cited the opportunity to personalize SMS text messages and the convenience of not having to log on to another platform as reasons for preferring SMS text messaging over other mHealth modalities [10]. Participants discussed which types of messages would resonate best with adolescents and how to present messages to maximize engagement. They suggested that messages promoting goal setting and the self-monitoring of behaviors as well as tailored content based on user characteristics and reported behaviors would allow for more participant engagement [10].

### The CBPR Process

The CBPR approach we used for the Teen HEED study involved the development of each aspect of the project with members of our community action board (CAB), including youths. For the mHealth component, we aimed to develop an SMS text messaging platform that would engage teens throughout the week and coincide with content from the weekly workshop sessions. Youth members of our CAB’s technology subcommittee (aged 16 to 22 years) used the results of focus groups conducted at an earlier time and published separately to brainstorm message types and frequency as well as to draft content [14]. We iteratively developed messages with the clarification of content queried by email and phone to gather further youths’ feedback. Their input contributed to the language used in automated SMS text message prompts and system responses, including words of positive reinforcement and motivational graphics or images. We collaboratively developed 5 categories of messages and created a spreadsheet with weekly messages, including the day and time of message delivery and automated responses to messages received (refer to Table 1 for the descriptions and examples of each message type). Goal-setting messages prompt participants to enter a weekly goal, report their progress by midweek, and report whether they completed their goal at the end of the week. Repeated behavior-tracking messages focus on key dietary behaviors (fruit or vegetable and sugary drink intake) and physical activity behaviors (moderate to vigorous physical activity and screen time). Workshop-specific messages focus on behaviors covered in the session that week (eg, reading nutrition labels, plate planning, and incorporating activity into daily routines). Tailored messages ask participants questions about their habits, with individualized feedback or guidance given based on their responses. Motivational messages are inspirational quotes or graphics. Finally, photo diary messages ask participants to “snap a pic” of different things related to healthy lifestyle to upload later in the week.

**Table 1:** Message type, description, and examples.

Message type	Description	Examples
Goal setting	Participants are prompted to enter a weekly goal, check in regarding progress, and report on goal completion.	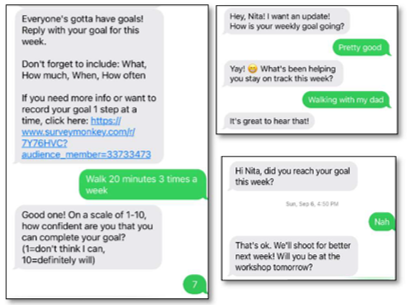
Behavior tracking	Participants report on behaviors (eg, reading nutrition labels) and record key behaviors over time (fruit and vegetable intake, screen time, physical activity, and servings of sugary beverages) with weekly individualized feedback.	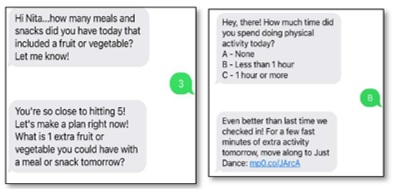
Tailored	Participants receive automated messages with feedback based on responses to questions.	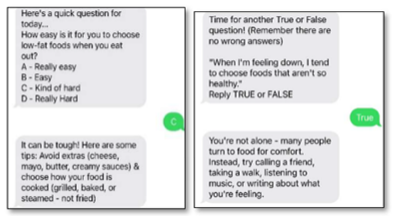
Motivational	Inspirational quotes and links to motivational graphics with text are sent to participants.	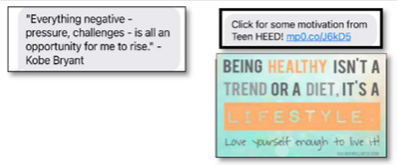
Photo diary	Participants are prompted to upload images pertaining to healthy eating and active living.	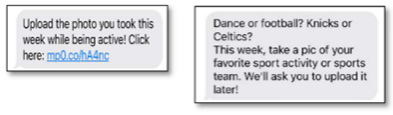

### mPulse Mobile Platform

We developed an SMS text messaging program with our partner technology company mPulse Mobile (HITRUST Common Security Framework [HITRUST Alliance] certified as well as compliant with Health Insurance Portability and Accountability Act standards). User profiles are entered into the system with basic demographic information. The *About* section of the participant’s profile is updated based on weekly responses to message prompts. We programmed message content developed by our CAB’s technology subcommittee into workflows on the mPulse Mobile platform, which is based on interactive dialogues (automated rule-based branching logic that allows users to navigate through the program based on their message responses). We collaboratively and iteratively refined how the youth-developed content was programmed through weekly meetings. Some early refinements included adding no-response reminders to goal-setting and behavior-tracking dialogues, reordering messages so that participants receive an average of 2 messages per day, balancing interactive and 1-way messages, streamlining the goal-setting prompt 4 weeks into the program, adding workflows for a more interactive experience, and modifying messages for the participants who did not attend the workshop sessions. An innovative program feature is artificial intelligence (AI) or natural language understanding (real-time interpretation of text responses that enables more sophisticated dialogues and solution-specific conversational agent capabilities). Beyond processing the responses that move users through automated dialogues, natural language understanding and a library of automatic replies handle user responses that fall outside the scope of the dialogue’s rules. An engagement console allows research staff to manage individual interactions with participants. The console identifies responses that cannot be handled by the messaging program’s rules, allows staff to manually initiate messages, and flags high-priority messages for escalation in real time for review and response by project staff.

### Internal Pilot Testing

After the development of the prototype, we conducted an internal pilot with 13 testers, including the principal investigator, the research coordinator, members of our CAB technology subcommittee, and mPulse Mobile team members. We held weekly meetings to discuss what was working and suggest improvements. After testing the content from weeks 1 and 2, we decided to decrease the message frequency by reducing the number of behavior-tracking messages from 4 to 2 per week and alternating dietary and physical activity questions so that each question would be asked every other week. We also reduced the motivational and “snap a pic” messages from 2 to 1 per week. Once our program developer made these changes, the internal pilot restarted from week 1 to evaluate these changes. Additional changes made during internal pilot testing included limiting morning messages to noninteractive dialogues, introducing a *helper bot* to automate responses when the system did not understand a user’s response, removing photo upload messages that included user’s faces to maintain anonymity, adding a motivational message halfway through the program, changing links that did not work, creating a global rule for *Not sure* or *Don’t know* responses, and adjusting message timing.

### Pilot Testing With Youths

We next invited youths from our pediatric clinic and the East Harlem community aged 13 to 21 years who had previously participated in the Teen HEED study to pilot the SMS text messaging program. The study coordinator individually contacted 26 previously enrolled study participants by phone and email to gauge their interest in pilot-testing the texting program. Ultimately, 13 youths consented (n=9, 69% who had been randomized to the intervention group and previously attended our in-person workshops and n=4, 31% who had been randomized to the control group and had not attended the workshops), and they participated in the 12-week pilot from August 2020 to November 2020. Participants received 2 system-automated messages per day at 9 AM, 3 PM, 6 PM, or 9 PM (the timings varied based on message type). The timing of messages was intentional as we discussed when youths were more likely to be available to respond to interactive messages and when different messages were most relevant (eg, adolescents were most likely to be tempted or challenged with unhealthy behaviors after school and to reflect on their behaviors at the end of the day). To obtain additional information about message content and optimal message frequency, we decreased the number of messages sent in weeks 8 to 11 and included some repeat messages from previous weeks. In week 12, participants received a final congratulatory message for completing the program. At the end of the 12 weeks, the participants took part in a focus group via Zoom (Zoom Video Communications, Inc) during which we asked about general system feedback, message frequency, message types and content, and suggestions for improvement.

### Data Collection and Analysis

Participant engagement data were collected by the SMS text messaging platform and viewable in the mPulse Mobile engagement console. The SMS text messages often asked questions or prompted users to reply; for example, a goal-setting prompt (“Would you like to set a goal today?”) would expect a response. The platform would *listen* for these responses and follow-up with an automated message in real time using rules and natural language processing. Every message sent and received by the system was time stamped and saved in the platform and linked to a participant’s unique ID. In addition to automated messages generated by the platform, the engagement console was occasionally used to reply to participants directly with more personal and nonautomated messages. If a participant replied to a 2-way interactive SMS text message, they were considered to have engaged; and if they did not reply to a 2-way interactive SMS text message, they were considered to have not engaged. If they replied “stop” to opt out, they were considered to have opted out. Participant demographics were collected via self-administered surveys. All quantitative data from texting outreach and participant demographics were analyzed using R statistical software (R Foundation for Statistical Computing) and Excel (Microsoft Corp), and data visualization was conducted using Tableau (Tableau Software, LLC). We analyzed qualitative data from the focus group using notes from the moderator and verbatim transcription of participant quotes from the focus group audio recording. We used open coding and thematic analysis to identify key themes that will inform planned program adaptations.

### Ethical Considerations

This study was approved by the institutional review board at the Icahn School of Medicine at Mount Sinai (STUDY-14-00359). We obtained informed consent from adolescents aged ≥18 years and from parents or caregivers for adolescents aged <18 years. No compensation was provided to the participants for this portion of the study. Study data, which were deidentified to protect participant privacy and confidentiality, were secured on password-protected Icahn School of Medicine at Mount Sinai servers.

## Results

### Participants

Descriptive statistics for the youths who participated in the SMS text messaging pilot are presented in Table 2. Participants ranged in age from 15 to 21 (mean 18.54, SD 1.87) years and most of them (10/13, 77%) identified as female. In terms of race and ethnicity, 23% (3/13) of the participants identified as Black, and 77% (10/13) identified as Hispanic or Latinx. More than half of the participants (7/13, 54%) had parents who were born outside the United States. Of the 9 participants who had previously taken part in the in-person workshop, 2 (22%) had attended <4 sessions, 3 (33%) had attended 5 to 8 sessions, and 4 (44%) had attended 9 to 12 sessions.

**Table 2:** Descriptive statistics.

Demographics		Values, n (%)
**Sex (n=13)**		
	Male	3 (23)
	Female	10 (77)
**Race and ethnicity (n=13)**		
	Black or African American	3 (23)
	Hispanic or Latino	10 (77)
**Type of Hispanic or Latino (n=10)**		
	Ecuadorian	1 (10)
	Honduran	2 (20)
	Mexican	3 (30)
	Puerto Rican	4 (40)
**Parent place of birth (n=13)**		
	Caribbean	1 (8)
	Ecuador	1 (8)
	Honduras	2 (15)
	Mexico	3 (23)
	Puerto Rico	1 (8)
	United States	5 (38)
**In-person workshop group (n=13)**		
	Intervention	9 (69)
	Control	4 (31)
**Session attendance for intervention group (n=9)**		
	0-4	2 (22)
	5-8	3 (33)
	9-12	4 (44)
**Virtual Teen HEED^a^ activity (n=13)**		
	1 (little to no response)	2 (15)
	2 (slightly responsive)	0 (0)
	3 (somewhat responsive)	4 (31)
	4 (fairly responsive)	1 (8)
	5 (highly responsive)	6 (46)

^a^HEED: Help Educate to Eliminate Diabetes.

We ranked participants based on their level of engagement (responsiveness to the 2-way interactive messages), with 15% (2/13) responding 0% to 10% of the time, 31% (4/13) responding 25% to 50% of the time, and 46% (6/13) responding >75% of the time over the 12 weeks. We analyzed responses from participants to SMS text messages about their typical diet and physical activity behaviors. Of the participants who responded to the messages, most (4/6, 67%) stated that they found it hard to choose low-fat foods when they ate out, all (7/7, 100%) usually order a medium-size fast food meal, most (6/7, 86%) reported that they at times eat a bigger portion than what they need, most (7/8, 88%) enjoyed drinking water, most (8/9, 89%) did not drink juice with their breakfast, and most (5/7, 71%) reported that they tend to choose unhealthy foods when they are feeling down. When asked about physical activity, most (4/6, 67%) stated that they get some activity but could do more. Most (6/7, 86%) agreed that it was important for them to look their best (because they value appearance).

### Message Delivery and Level of Engagement

**Figure 2:**
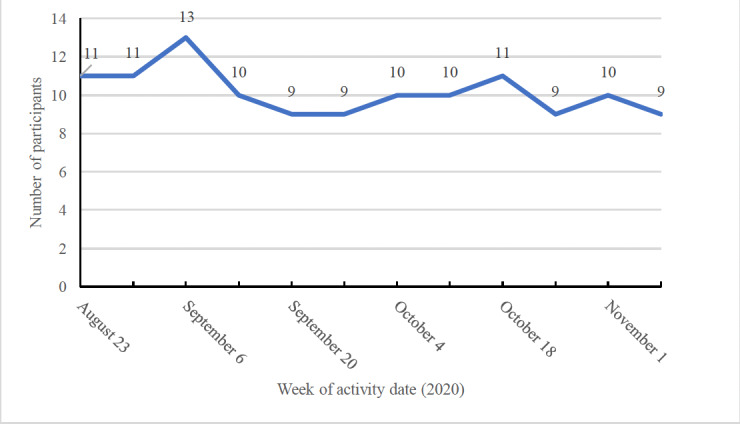
Engaged members by week.

When tracking the fidelity of message delivery, we found that 84% (2231/2656) of the messages were sent correctly (on the expected date and time). To address any errors in message delivery, we tracked the messages in the engagement console and manually sent any messages that were either not sent or sent incorrectly. Over the 12 weeks of the pilot program, 85% (11/13) of the participants were responsive to the interactive 2-way messages, with 69% (9/13) still engaged with the program at week 12 (Figure 2). The number of interactive messages sent and received were recorded monthly and are presented in Textbox 1. Messages were labeled as 2-way (or interactive) if they ended in a question mark and a response was expected. Response rates to the interactive messages started at almost 60% (76/134) in the first month and decreased to approximately 30% (54/198) toward the end of the program when participants received fewer messages from the system. The system received an average of 77 messages per week (7 messages/active user/week) over the first 7 weeks of the program and an average of 47 messages per week (approximately 4 messages/active user/week) in weeks 8 to 12 when we reduced the number of weekly messages sent.

In addition, we originally programmed the system to send a response to any messages received (a programmed response; an emoji; an encouraging statement; or, in case the system could not understand a response, a variation of “Sorry, but I’m just a bot so I don’t have a clever response. 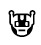 Remember, you can reach out to Dr. Nita directly if you need!”). When tracking messages in the engagement console, we noticed that some users engaged less with the system after receiving multiple bot messages. As a result, we opted to remove the bot messages after week 5 and instead had the project coordinator monitor the console and manually respond to participants when necessary.

**Participant interaction with message types sent.**

**Messages sent by type**

- August
  - 2-way: 134
  - 1-way: 97
- September
  - 2-way: 603
  - 1-way: 551
- October
  - 2-way: 603
  - 1-way: 396
- November
  - 2-way: 198
  - 1-way: 74

**Monthly engagement rate for 2-way interactive messages (messages received/messages sent)**

- August: 56.7% (76/134)
- September: 39.8% (240/603)
- October: 31.5% (190/603)
- November: 27.3% (54/198)

### Focus Group Findings and Planned Adaptations

In addition to tracking the engagement console and user analytics as described previously, we conducted a focus group with 11 (85%) of the 13 participants after the 12-week program to further examine user feedback about message timing, frequency, type, and content. We asked participants to choose individual words to describe the program, and the responses included “interactive, helpful, informative, comforting, enjoyable, convenient, reliable, motivational, thoughtful, healthy, productive, and reflective.” When asked about the number of messages, participants remarked that the messages “were not intruding...considering that the program is only through messages, I think the frequency of the messages is necessary, so that [participants] feel the presence—that the program is there.” When the frequency of messages was reduced after week 7, participants reported that they noticed the decrease in frequency, felt that the program was coming to a close, and missed the additional messages. Those who had also participated in our in-person workshops mentioned that the SMS text messages were reminiscent of the in-person workshop content. Overall, participants enjoyed goal setting, behavior tracking, and personalized messages the most. With busy school and work schedules, participants appreciated receiving multiple messages to review their goals and track their progress each week. They suggested that messages could be further tailored based on their user profile and personal habits or goals. A summary of relevant quotes and planned program adaptations informed by the focus group findings is presented in Table 3.

**Table 3:** Planned adaptations to the SMS text messaging platform informed by the focus group findings.

Findings from pilot testing			Planned adaptations to platform
**Message frequency**			
	“You felt like the program was coming to an end once the messages started coming less frequently. It was a little sad since I was doing it since school started so it was part of my routine.”	Maintain the same message frequency throughout program, with less frequent messages only after workshop completion (an average of 2 daily messages in the first 12 week followed by an automated weekly maintenance message and additional manually initiated messages through the 12-month follow-up).	
**Tailored messages**			
	“It served to remind me of the things I could be doing...These types were the best for me when I was very busy because they were very quick...they’re simple, short, quick facts.”	Personalize messages using data entered when creating user profile (personal characteristics, habits, and goals).	
	“If it was a little more interactive, I would have benefited more from it, instead of responding and forgetting about it.”	Increase interactivity with more 2-way exchanges and detail in dialogues.	
**Goal setting**			
	“I think they were great. I just wish they were more repeated at the date and time that I chose to set my goal for.”	Use natural language understanding and peer leader messaging through the engagement console for the personalization of goal reminders.	
	“Maybe [the system should] ask ‘What troubles did you have?’ and then after you respond it should follow up with ‘Would you like to talk with a peer mentor?’—if you say yes, it will schedule one, and if you say no you can continue on with your day.”	Hybridize automated messaging and human interactions to troubleshoot challenges with goal completion.	
**Behavior tracking**			
	“The frequency, the variety of these types of messages are good as is. I got a link to Just Dance, but I have to be honest, I didn’t press on the link mainly because of the timing of those messages. I think it was always while I was on the bus coming home from work, and the last thing I really wanted to do was watch a video.”	Personalize messages using data entered when creating user profile (personal characteristics, habits, and goals), and customize message timing based on user schedules.	
**Workshop-specific messages**			
	“I agree that the messages of MyPlate reminded me of activities from the actual program.”	Increase the frequency of messages reflecting workshop content.	
	“Because they were short, I liked them. They did remind me of the workshop. These are good for small reflections.”	Increase the frequency of messages reflecting workshop content.	
**Motivational messages**			
	“I liked a good amount of the linked ones...but the quotes were just better and easier than the linked ones.”	Increase focus on inspirational quotations for motivation instead of links to graphics.	
**Photo diary**			
	“I thought it was going to be a more collaborative thing when we sent the photos. Without that, it feels like we’re sending the photos for nothing.”	Create a shared space for photos to increase interactions among participants.	
“**I’m just a bot” messages**			
	“I knew it was bound to happen because it’s a bot with automated messages. But I just tried to stop myself from saying random things because the bot doesn’t understand.”	Remove “I’m just a bot. I don’t understand” messages, refine artificial intelligence and natural language understanding capabilities, and monitor engagement console for instances when system cannot handle messages.	
**Peer leader–initiated messages**			
	“I noticed a difference in my response to Cordelia’s personal messages—I thought, ‘Oh, it’s a human, so I can actually respond and a person’s going to read it right then and there and possibly respond. So I made a more lengthy response—something more meaningful.”	Increase the hybridization of automated messages and peer leader–initiated messages for increased engagement.	
**Additional suggestions**			
	Quick statistics, facts, and tips, as well as short quizzes	Add brief messages focused on diabetes statistics, facts, tips, and quizzes about workshop content.	
	Group competitions	Increase social interaction with friendly competitions (step challenges, etc).	
	Visual content	Share group accomplishments visually and incorporate visual storytelling message content.	

## Discussion

### Principal Findings

Our SMS text messaging program was well received by participants based on both analytics and qualitative feedback. At least 9 (69%) of the 13 participants who initially consented to the study were engaged each week throughout the 12 weeks. In addition, we received 50 to 75 messages per week across the 13 users (a range of 4-8 messages received/user/week). Of note, we had high engagement rates despite having a higher dose of messages delivered weekly than other similar programs [15]. A systematic review of SMS text messaging interventions for weight management in adolescents found a median of 1.5 (range 1-21) SMS text messages sent per week across the studies, with half of the interventions including delivery of messages only once per week or once per month [15]. By contrast, we sent an average of 2 messages per day. Jensen et al [16] found that most participants would prefer fewer messages, but our participants seemed to miss the high volume of SMS text messages once the dose decreased in later weeks. One reason for this may have been the incorporation of messages focused on goal setting, behavior tracking, and tailored guidance, which our participants favored based on our focus group findings and which might have increased the relevance and perceived value of the messaging.

### Comparison With Prior Work

Among youth mHealth lifestyle interventions, SMS text messaging has been shown to be more acceptable and effective as an intervention delivery method than mHealth apps and social media [15,17]. A recent systematic review of the effectiveness of SMS text messaging interventions for weight management in adolescents found that 7 (88%) of the 8 identified studies demonstrated a decrease in BMI or BMI *z* score in the intervention group compared with the control group [15]. A 2012 Pew Research Center survey showed texting by far to be the most common daily form of communication used by teens, even at a time when only 77% of teens reported having a mobile phone [18]. These results align with what we learned from our prior focus groups with youths in which most teens preferred SMS text messaging as the delivery method to support our diabetes prevention program [14]. Teens preferred SMS text messaging over social media and mHealth apps owing to the ease of not having to log on to a separate platform and stated that they would be more likely to respond to an SMS text message than to a notification via Facebook or an app [14]. Given the strong evidence supporting SMS text messaging as a modality for delivering lifestyle interventions among youths, as well as the specific support for this strategy from the participants in our own focus groups, we chose to deliver our intervention through SMS text messaging.

Although there are examples of effective SMS text message–based lifestyle intervention studies in youths, our study addressed multiple gaps in the literature [19-21]. First, there is limited information about the most effective message content and timing [15,22-26]. In a systematic review of the effectiveness of SMS text messaging interventions for weight management in adolescents, Partridge et al [15] found that although most teens said that they preferred more personalized messages, only half of the SMS text messaging interventions for weight management in adolescents included features such as 2-way texting or personalized messages. The systematic review also found that adolescents who received interventions that incorporated 2-way interactive messages had better engagement than those in control groups and gained more knowledge than those who received 1-way messages. Newer technologies such as AI and natural language processing that have the ability to personalize SMS text messages and engage users in 2-way dialogues are only now starting to be incorporated into SMS text messaging programs [16,27-32]. We were able to harness the potential of these new features and technologies through our partnership with mPulse Mobile.

Another important aspect of our study that distinguishes it from prior studies is the level of involvement of youths as members of our study team throughout the process. Some studies have incorporated youths’ feedback through participation in focus groups or surveys after the development of SMS text messaging interventions; for example, Jensen et al [16] tested SMS text messages in a 3-month feasibility study by using surveys and semistructured interviews to garner feedback about message content and timing [16]. Two other studies sought youths’ perspectives on mHealth apps used for lifestyle interventions in youths with obesity without building an actual app [25,33]. Another study had an advisory team with 4 adolescents who gave formative feedback on content and delivery modality for their mHealth intervention, and youths who participated in the subsequent randomized controlled trial gave feedback through a 6-item multiple-choice questionnaire [20]. However, none of these studies involved youths as equal stakeholders throughout the development process [20,34,35]. The few prior studies that used more participatory approaches to develop message content for adolescents resulted in high engagement and acceptability [15]. In alignment with these studies, we partnered with local youths who were deeply involved in the development, testing, and evaluation of our program.

### Triple Partnership: Youths, Technology, and Academia

In addition, few mHealth studies have included diverse stakeholders, including academic researchers, technology experts, and members of the target population. To our knowledge, no prior study has leveraged the type of unique 3-way partnership we built to develop an mHealth lifestyle intervention for youths. Through our partnership with the youths on our CAB and mPulse Mobile, we are the first team to implement such a collaboration. Some previously cited reasons for the lack of academic-industry partnerships include, as described in the study by Hingle et al [36], “(1) (for the academic) striking the ‘right’ balance between cultivating a relationship with industry partners while navigating the practical considerations and constraints of academic appointments; (2) agreeing on ambitious but feasible timelines and deliverables to meet industry demands; and (3) selecting appropriate outcome and evaluation metrics that stand up to the rigor of academic research while remaining responsive to market-values and industry priorities.” For our study, it was important to find a technology partner who was not primarily motivated by how financially lucrative the developed platform would be so that we could first focus on developing the program collaboratively with our youth partners to make it appealing to our target population. The philanthropic nature of our initial collaboration has been the foundation for us to develop a mutually beneficial relationship because industry helps the community, the community informs industry, and we are able to use our expertise as researchers to evaluate and disseminate our work. In this way, each group can share their insights and benefit from the knowledge gained.

### Strengths and Limitations

The strengths of this study include the use of CBPR methods with the deep involvement of youths from our community in the creation and pilot testing of our SMS text messaging program. This novel program uses AI and natural language understanding features to provide tailored 2-way messages. This study is also unique in its triple partnership among academia, program developers, and racially and ethnically minoritized youths to address disparities in obesity and diabetes rates.

The limitations of the study include the small sample size for pilot-testing our program. Other studies that tested an SMS text messaging lifestyle intervention among youths had a wide range of sample sizes, from 14 to 47 participants [16,20,30,37]. Our findings are specific to the small group of youths we collaborated with to develop and pilot our program and should not be generalized to youths from populations that are significantly different from ours. Although our study included more female and Hispanic participants, we could not examine trends in these subgroups owing to the small sample size but plan to explore subgroup differences in future studies. Our study was conducted during the summer of 2020 at the height of the COVID-19 pandemic in New York City. This may have influenced the engagement of the participants. In addition, our participants included teens who were highly engaged in the original Teen HEED study and had strong relationships with the study team. Thus, the high engagement rates we observed may not be representative of what would be observed among larger groups of teens. We also had some unforeseen technical issues and limitations in the types of messages we could send; for example, we could not include SMS text messages with embedded images; therefore, our motivational messages instead had to focus on inspirational quotes and web links to images. Despite these limitations, the participants still enjoyed these messages.

### Conclusions and Future Directions

Our process, feasibility, and acceptability study informed several strategies to improve the SMS text messaging program for future iterations of Teen HEED, as summarized in Table 3. One such adaptation is implementing a hybridized model between human and autogenerated messages. One study in which all SMS text message reminders sent to participants were human-generated reported response rates of 70% to 80% [38]. In our study as well, we noted that participants responded more positively to messages sent manually by the project coordinator than to the bot-generated error messages. Although human-generated messages require additional time and labor, the possibility for increased engagement suggests that a hybridized model may be useful to increase engagement while remaining feasible. This will require more effort to implement, but we plan to prioritize certain participants and situations for this type of direct human interaction (eg, participants with low response rates, those who are not meeting their goals, or those who request additional support). In addition, participants suggested that the ability to share the experience with other youths would be helpful. This could be incorporated by sharing the photo journal upload results in a common space to create a sense of community. Other youth mHealth studies have focused on peer support to increase acceptability and engagement [34]. Friendly competitions and creating ways to share group accomplishments are another way to increase social interactivity and engagement. Finally, in the next iteration of Teen HEED, we plan to maintain the same message frequency throughout the program and to further customize messages based on user profiles and trends in reported behaviors over time.

We hope that the results from this study can inform future SMS text messaging programs targeting youths to address health disparities.

## Abbreviations

AI: artificial intelligence
CAB: community action board
CBPR: community-based participatory research
CONSORT: Consolidated Standards of Reporting Trials
HEED: Help Educate to Eliminate Diabetes
mHealth: mobile health
T2DM: type 2 diabetes mellitus

## Data Availability

The data sets generated and analyzed during this study are not publicly available owing to the small sample size and potential for our participants to be identified. However, the data set is available from the corresponding author on request.
